# Transcriptome analysis of the bivalve *Placuna placenta* mantle reveals potential biomineralization-related genes

**DOI:** 10.1038/s41598-022-08610-5

**Published:** 2022-03-18

**Authors:** Ningjing Song, Jiangfeng Li, Baosheng Li, Ercai Pan, Yurong Ma

**Affiliations:** School of Chemistry and Chemical Engineering, Beijing Institute of Technology, Beijing, 100081 China

**Keywords:** PCR-based techniques, Transcriptomics, Comparative genomics

## Abstract

The shells of window pane oyster *Placuna placenta* are very thin and exhibit excellent optical transparency and mechanical robustness. However, little is known about the biomineralization-related proteins of the shells of *P. placenta*. In this work, we report the comprehensive transcriptome of the mantle tissue of *P. placenta* for the first time*.* The unigenes of the mantle tissue of *P. placenta* were annotated by using the public databases such as nr, GO, KOG, KEGG, and Pfam. 24,343 unigenes were annotated according to Pfam database, accounting for 21.48% of the total unigenes. We find that half of the annotated unigenes of the mantle tissue of *P. placenta* are consistent to the annotated unigenes from pacific oyster *Crassostrea gigas* according to nr database. The unigene sequence analysis from the mantle tissue of *P. placenta* indicates that 465,392 potential single nucleotide polymorphisms (SNPs) and 62,103 potential indel markers were identified from 60,371 unigenes. 178 unigenes of the mantle tissue of *P. placenta* are found to be homologous to those reported proteins related to the biomineralization process of molluscan shells, while 18 of them are highly expressed unigenes in the mantle tissue. It is proposed that four unigenes with the highest expression levels in the mantle tissue are very often related to the biomineralization process, while another three unigenes are potentially related to the biomineralization process according to the Quantitative Real-Time Polymerase Chain Reaction (qRT-PCR) analysis. In summary, the transcriptome analysis of the mantle tissue of *P. Placenta* shows the potential biomineralization-related proteins and this work may shed light for the shell formation mechanism of bivalves.

## Introduction

The biological exoskeletons are usually composed of highly ordered hierarchical micro- and nanostructures occluded with organic molecules and exhibit superior mechanical, optical, thermal, magnetic properties ^1–3^. Calcium carbonate is one of the most abundant biominerals in nature, and the biomineralization and bioinspired mineralization of calcium carbonate have been the focus of investigation for many decades. The molluscan shells have well defined micro- and nanostructures and excellent mechanical properties, composed of 95% CaCO_3_ and less than 5 wt % of organic macromolecules (proteins, glycoproteins, polysaccharides and lipids) ^4,5^. Generally, the polymorphs of CaCO_3_ include aragonite, calcite, vaterite, calcite monohydratre and calcium carbonate hemihydrate ^6^. Biogenic CaCO_3_ exhibits complex microstructures such as prismatic, nacreous, foliate, and cross-lamellar microstructures ^7,8^. Importantly, protein components in the molluscan shells are responsible for the nucleation and crystal growth of biogenic CaCO_3_ and can enhance the mechanical properties of the shells ^9^. The matrix proteins occluded in the molluscan shells were extracted, sequenced and identified as biomineralization-related proteins which were found to have functions including stabilization of amorphous calcium carbonate, inhibition or acceleration of nucleation of calcium carbonate ^10–13^. A few examples of biomineralization-related proteins include mantle protein N25 ^14^ , perlucin, perlustrin ^15^, aspein ^16^, prisilkin-39 ^17^, Shematrin-2 ^18^, PfN44 ^19^, and enzymes such as carbonic anhydrase ^20^. Most of matrix proteins in molluscan shells are specifically secreted by the mantle tissue of molluscans. In another word, the proteins from the mantle tissues of molluscans can tune the formation of different structural layers of the shells ^12,21,22^. It was considered that the shell matrix proteins extracted from skeletons or shells generally have high expression in mantle tissues. Identification and characterization of the proteins that are involved in the biomineralization process are critically important for understanding formation mechanisms of shells. Biomineralization-related proteins have been reported from the bivalve species such as perlucin from abalone *Haliotis laevigata* ^23^, nacrein from pearl oyster *Pinctada fucata* ^24^, and aspartic acid rich proteins from *Atrina rigida* ^25^.

*P. placenta*, also known as window pane oyster, has two highly transparent, disc-shaped flat mineralized valves. Local people used to apply the shells of *P. placenta* as a substitute for window glass in houses in the old days. *P. placenta* live at the coasts of East China Sea and South China Sea. *P. placenta* are often found in sandy or muddy substrates in shallow estuarine lagoons and small bays. The inner surface of the *P. placenta* shell has a V-shaped ligament. The right valve is almost flat while the left valve is slightly convex ^26^. The *P. placenta* shell is composed of foliated calcite single crystals with thickness of about 300 nm. The *c* axis of these calcific laths is tilted about 24.4º ± 3.5º from the normal direction of the shell surface. Thus, the surfaces of the laths are close to the {108} planes of calcite ^27,28^. In addition, the shell of the *P. placenta* possesses unique transparent optical property, which can transmit ~ 80% of visible light. The hardness of the shell of the *P. placenta* increases ∼55% in comparison to that of single-crystal calcite ^29,30^.

It was considered that the shell matrix proteins extracted from skeletons or shells generally have high expression in mantle tissues. The transcriptome is the complete set of transcripts in the tissues of organisms, which contains important information on gene expression and contributes to understand the functional components of the transcriptome and molecular constituents of cells and tissues ^31^. Therefore, the study of molluscan mantle transcriptome is important for the identification of biomineralization related proteins, which can provide further understanding for the shell formation mechanism. Mantle transcriptome studies from different molluscan shell systems such as trochidae *Tectus pyramis* ^32^, freshwater pearl mussel *Cristaria plicata* ^33^, pearl oyster *Pteria penguin* ^34^, *Pinctada martensii* ^35^, scallop *Patinopecten yessoensis* ^36^ and *Chlamys farreri* ^37^ have been investigated for the purpose of identifying biomineralization related genes. However, high-throughput sequencing and the transcriptome-based identification of potential biomineralization unigenes of the mantle tissue of *P. placenta* transcriptome have not been reported, as far as we know.

Recently, a new generation efficient sequencing technology has been developed for transcriptome study by using Illumina platform. In comparison to traditional methods, Illumina sequencing has advantages such as low cost, high-throughput, and rapid sequence generation. Thus it has been applied as an effective way to identify potential unigenes or pathways involved in the processes of biomineralization in molluscans ^37–39^.

Herein, we report the mantle transcriptome for *P. placenta* by Illumina-based RNA-Seq technology for the first time. Dataset of the transcripts were annotated by using five different public databases. Potential single nucleotide polymorphisms (SNP), indel markers and simple sequence repeat (SSR) markers were analyzed based on the transcriptome dataset of the mantle tissue of *P. placenta*. Unigene sequences, pfam analysis and qRT-PCR analysis were done for the highly expressed unigenes of the mantle tissue and several potential genes related to biomineralization were identified.

## Materials and methods

### Animals

Adult wild *P. placenta* were two years old and collected during July to August (2020) from Beibu Gulf (Fangchenggang city, Guangxi province, China). The shells were almost-flat and had diameters in between 50 and 70 mm. Different tissues such as mantle, adduction muscle, gonad, gill, hepatopancreas, mouthparts, and intestine were dissected from fresh adult wild *P. placenta* and were frozen immediately in liquid nitrogen, then stored at -80 ºC for further analysis. All animal experiments were done in accordance with the guidelines and approval of the Animal Research and Ethics Committees of the Chinese Academy of Sciences.

### Materials

RNA Keeper-ICE tissue transfer buffer was bought from vazyme company (Nanjing, China). Quick RNA Isolation kit was bought from Biotech Corporation (Beijing, China). AMPure XP beads were bought from Beckman (CA, USA). HiScript® II Q RT SuperMix reagent was bought from vazyme company (Nanjing, China). TRIzol reagent was bought from TransGen Biotech (Beijing, China). Divalent cation fragmentation buffer was bought from Illumina (CA, USA). RNaseH and DNA polymerase I were bought from TaKaRa (Kyoto, Japan). Elution buffer was bought from GENEWIZ (Suzhou, China). Agarose gel electrophoresis buffer was obtained by dissolving 1 g of agarose in 100 mL of Tris Acetate EDTA electrophoresis buffer. Tris Acetate EDTA electrophoresis buffer was bought from TSINGKE (Beijing, China). Agarose was bought from Sigma (Darmstadt, Germany).

### Total RNA extraction, cDNA library preparation and Illumina sequencing

Total RNA was extracted from the mantle tissue of *P. placenta* by using the Quick RNA Isolation Kit (Biotech Corporation, Beijing, China). The concentrations of RNA were quantified with a Qubit 2.0 Fluorometer (Invitrogen, Life Technologies, CA, USA), and the RNA integrity was evaluated using an Agilent 2100 Bioanalyzer (Agilent Technologies, Santa Clara, CA, USA). The weight of the mantle tissue for RNA extraction was no less than 300 mg for each sample and the experiments were repeated for three times.

The cloned DNA (cDNA) library construction and sequencing analysis were performed at Beijing BioMarker Technologies (Beijing, China). The cDNA library was constructed as follows. In the case of eukaryotes, polyadenylation (poly (A)) tail messenger RNA (mRNA) was purified with oligonucleotides (dT) magnetic beads from total RNA, and then the mRNA-enriched RNA was randomly segmented into small fragments in a divalent cation fragmentation buffer (Illumina, Hayward, CA). These short fragments were used as templates to synthesize the first strand cDNA using random hexamer primers. The second strand cDNA was generated using RNaseH and DNA polymerase I. The double stranded cDNA was purified by beads (Beckman, CA, USA) and washed with elution buffer for end repairing and tailing A. Afterwards, these short fragments were ligated to sequencing adapters according to Illumina’s protocol (SanDiego, CA, USA) and were then separated by agarose gel electrophoresis.

In order to obtain the cDNA library, suitable fragments (300–500 bp) were selected as templates for PCR amplification. The effective concentration of the cDNA library (> 4nM) was accurately quantified by the quantitative real-time PCR (qRT-PCR) method. Finally, the cDNA libraries were sequenced on an Illumina HiSeq^TM^ 4000.

### De novo assembly and sequence annotation

Before de novo assembly, raw data were saved in fastq format and filtered for downstream analysis using in-house pear script, which mainly contains three filter steps: (1) discard sequencing adapter; (2) remove reads with more than 10% of unknown bases; (3) filter low-quality reads which contain more than 50% of low-quality bases (Q ≤ 20). After the above filtering processes, de novo assembly was carried out using Trinity with min_kmer_cov set to 3 by default and all other parameters set to their default values ^40^.

The coding regions of the assembled unigenes also known as the coding DNA sequence (CDS), were predicted by two steps. First, the unigene sequences from the mantle tissues of *P. placenta* were successively translated and compared to the NCBI non-redundant protein sequences (nr database) and a manually annotated and reviewed protein sequences (Swiss-Prot) database. Then, the Basic Local Alignment Search Tool (BLAST) analysis was used to annotate the functions of the protein sequences based on nr and Swiss-Prot databases with a cut-off E-value of 10^–5^. The protein sequences are actually amino acid sequences of proteins. These two proteins database have correct sequence to predict the coding regions of the unigenes. Second, The CDSs were determined using ESTScan with default settings if the unigenes could not be aligned to any entry of the above mentioned two databases ^41^. ESTScan software was used to determine the open reading frame and obtain the nucleic acid and amino acid sequences from the unannotated unigenes. Finally, all annotated unigenes were aligned to the public databases, including the euKaryotic Ortholog Groups (KOG, http://www.ncbi.nlm.nih.gov/COG/), Kyoto Encyclopedia of Genes and Genomes (KEGG, http://www.genome.jp/kegg/), Gene Ontology (GO, http://www.geneontology.org/) and Protein family (Pfam, http://pfam.sanger.ac.uk/) databases.

### SNP, Indel, SSR markers identification and primer design

Single nucleotide polymorphisms (SNP) refers to genetic markers formed by mutations of the single nucleotide on the genome. There are a large number of SNPs with abundant polymorphisms. Generally, SNP is defined as single nucleotide mutations with a mutation frequency more than 1%. Indel refers to the insertion and deletion of small fragments in the sample genome relative to the control genome. The small fragments may contain one or more bases. We identified SNPs and indels by using samtools, picard-tools and GATK2 software ^42^.

Simple sequence repeat (SSR), also known as short tandem repeats and microsatellite markers, which contains the number of repeat motifs with less than six nucleotides (di-, tri-, tetra-, penta-, and hexa-nucleotide). The repeat motifs exist often in the genome of eukaryotes. Unigenes sequences were used to search for potential SSR markers using MISA software (version 1.0, http://pgrc.ipk-gatersleben.de/misa/misa.html) with the default parameters. Afterwards, Primer 3 software (version 2.3.5, http://fokker.wi.mit.edu/primer3/ default parameters) was applied for SSR primer design.

### Identification of unigenes involved in biomineralization and comparison of transcriptomes

Biomineralization-related genes have been reported in the mantle tissues of molluscans such as prismalin-14 ^43^, pif ^44^, amorphous calcium carbonate binding protein ^45^, and asporin ^46^. Identification of the potential biomineralization-related unigenes were carried out by keyword searching according to the reported expressed unigenes in the nr database which were reported to be related to biomineralization. In general, three kinds of unigenes were considered as potential biomineralization-related unigenes, well-known unigenes that have been reported to play important roles for the shell formation, the reported unigenes which are highly homologous with the reported shell formation related unigenes, the proteins those were found in mollusk shells and had high expression in the mantle tissues of molluscan animals. All of the above-mentioned unigenes may probably play key roles for the shell formation. If several unigenes were assigned with the same reference gene, the unigene with the lowest E-value and highest score was assumed to be the homolog of the reference gene.

To compare the similarities and differences between the mineralization proteins of various molluscan animals, the transcriptome annotations of *P. placenta* were compared with the mantle transcriptome annotations from two other molluscans, *P. yessoensis* ^36^ and *C. farreri* ^37^.

### Pfam database analysis

Pfam is one of the most comprehensive classification systems for protein domain annotation ^47^. Biomineralization-related unigenes can be found from the Pfam database analysis of the *P. placenta* mantle according to the characteristics of domains found in molluscan shell matrix proteins (SMPs). The Hidden Markov Models (HMMs) of protein domains were obtained from Pfam. The HMMs were applied to study the protein domains and the unigene annotations by using HMMER3 program ^48^.

### Quantitative real-time polymerase chain reaction (qRT-PCR) analysis

In order to determine the expression levels of the potential biomineralization-related genes in different tissues of *P. placenta*, the unigenes from seven candidate tissues were characterized by using qRT-PCR. Seven sets of tissue samples were dissected from wild type of adult *P. placenta*, with shell sizes ranging from 50 to 70 mm. All the soft tissue including adduction muscle, gonad, gill, mantle, hepatopancreas, mouthparts, and intestine were dissected from fifteen fresh animals. For qRT-PCR measurement, the weight of each kind of tissue is no less than 300 mg. Each qRT-PCR analysis was replicated for three times. These tissues were kept in RNA Keeper-ICE tissue transfer buffer for RNA extraction. RNA Keeper-ICE tissue transfer buffer penetrates into the tissues during the defreezing process and the RNase of the tissues were inactivated. Total RNA from the different tissues were extracted with TRIzol Reagent at a ratio of 1 mL per 100 mg tissue. The concentration and purity of RNA were quantified using NanoDrop® ND-2000 (Thermo scientific). The RNA integrity was evaluated by Bioanalyzer 2200 (Agilent). The primer sequences used for qRT-PCR are listed in Table S1. 2 μg sample of isolated RNA was used to synthesize first-strand cDNA using HiScript® II Q RT SuperMix for qRT-PCR (Vazyme). PCR reactions were performed on an ABI 7900 RealTime PCR Detection System (Applied Biosystems, USA). The 10 μL qRT-PCR reaction mixture consisted of 5 μL of 2 × master mix, 1 µL of cDNA, and 0.4 µL primers. The actin gene was used as the reference. The qRT-PCRs were performed under the following conditions: heating at 95 °C 5 min, at 95 °C for 15 s for 40 cycles, at annealing temperature 60 ℃ for 1 min, and at 95 °C for 15 s, followed by melting curve detection at 60–95 °C. The comparative CT method was used to determine relative mRNA abundance ^49^.

## Result

### Sequence analysis and de novo assembly

A total of 29,996,311 raw reads were obtained after Illumina sequencing analysis. Considering that the raw reads contain a small amount of sequencing adapters and low-quality reads, the resulting clean reads were obtained after quality filtration and then subjected to de novo assembly. Firstly, the quality of the extracted RNA is in A grade (RIN > 7, for our data). Secondly, the Q20 percentage and Q30 percentage are 98.54% and 95.94%, respectively, and the error rate is 0.01%, which indicate that RNA sequencing results are qualified and reliable. The percentage of GC content for the clean reads is 44.45% (Table 1). These results show that the obtained unigenes are suitable for annotation.

**Table 1:** Statistics of Illumina sequencing in the mantle transcriptome of *P. placenta.*

Raw reads	29,996,311
Raw bases	4.49G
Clean reads	28,963,193
Clean bases	4.34G
Error rate	0.01%
Q20	98.54%
Q30	95.94%
GC Content	44.45%

**Figure 1:**
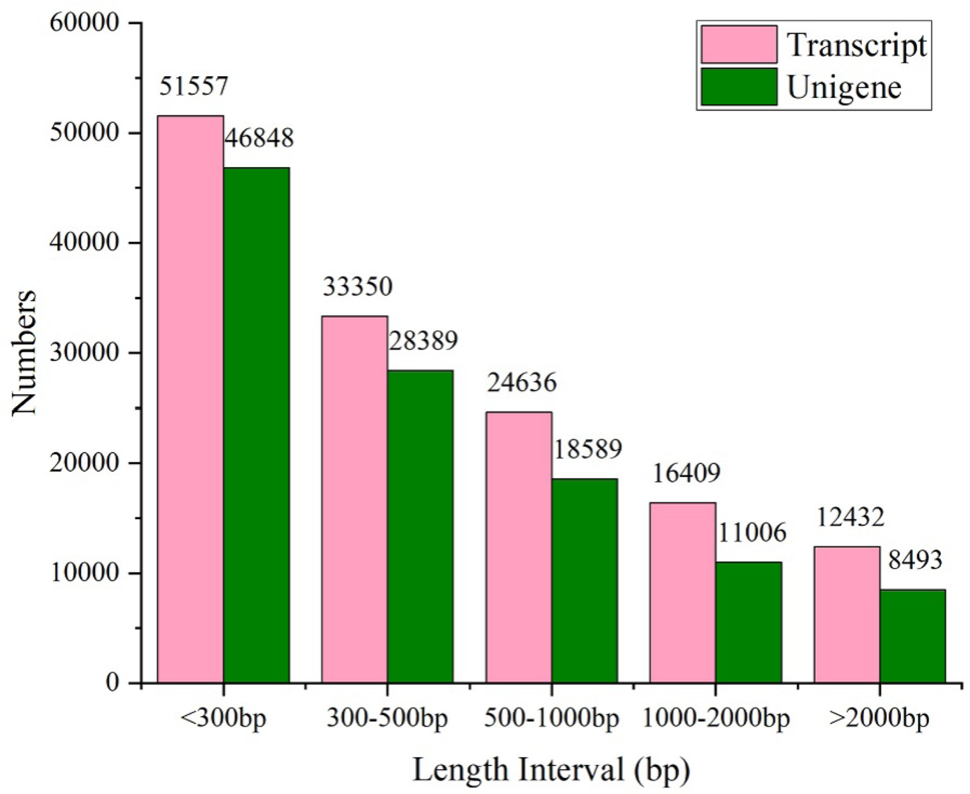
Sequence length distribution of transcripts and unigenes for the mantle transcriptome of *P. placenta* assembled from Illumina reads*.*

Filtered reads are assembled into 138,384 transcripts, with a total length of 106,972,212 nucleotides. These transcripts are subsequently assembled into 113,325 unigenes and the mean length of transcripts is 697 nucleotides, ranging from 201 to 39,605 nucleotides. N50 length is defined as the unigenes length L, while 50% of all bases in the sequences are in contigs of length less than L. The mean length of unigenes is 773 nucleotides, with N50 of 1,437. In summary, the total length of all unigenes is 79,007,878 nucleotides or 73.9% of the length of all transcripts (Table 2). The length-frequency distribution for unigenes and transcripts shows that the numbers of transcripts and unigenes decline with the increase of length, while the ratios of unigenes to transcripts with the same length ranges remain almost the same. The number of the unigenes with length less than 500 bp is 76,237, accounting for 66.39% of all unigenes, while less than one-third of long reads (greater than 1000 bp) are assembled into unigenes, indicating that short reads are more likely to be assembled into unigenes (Fig. 1).

**Table 2:** Statistics of de novo assembly for the mantle transcriptome of *P. placenta.*

Items	Total number	Total length (nt)	Mean length (nt)	Shortest length (nt)	Median length (nt)	Longest length (nt)	N50
Transcripts	138,384	106,972,212	773	201	378	39,605	1437
Unigenes	113,325	79,007,878	697	201	345	39,605	1249

### Functional annotations of unigenes

**Figure 2:**
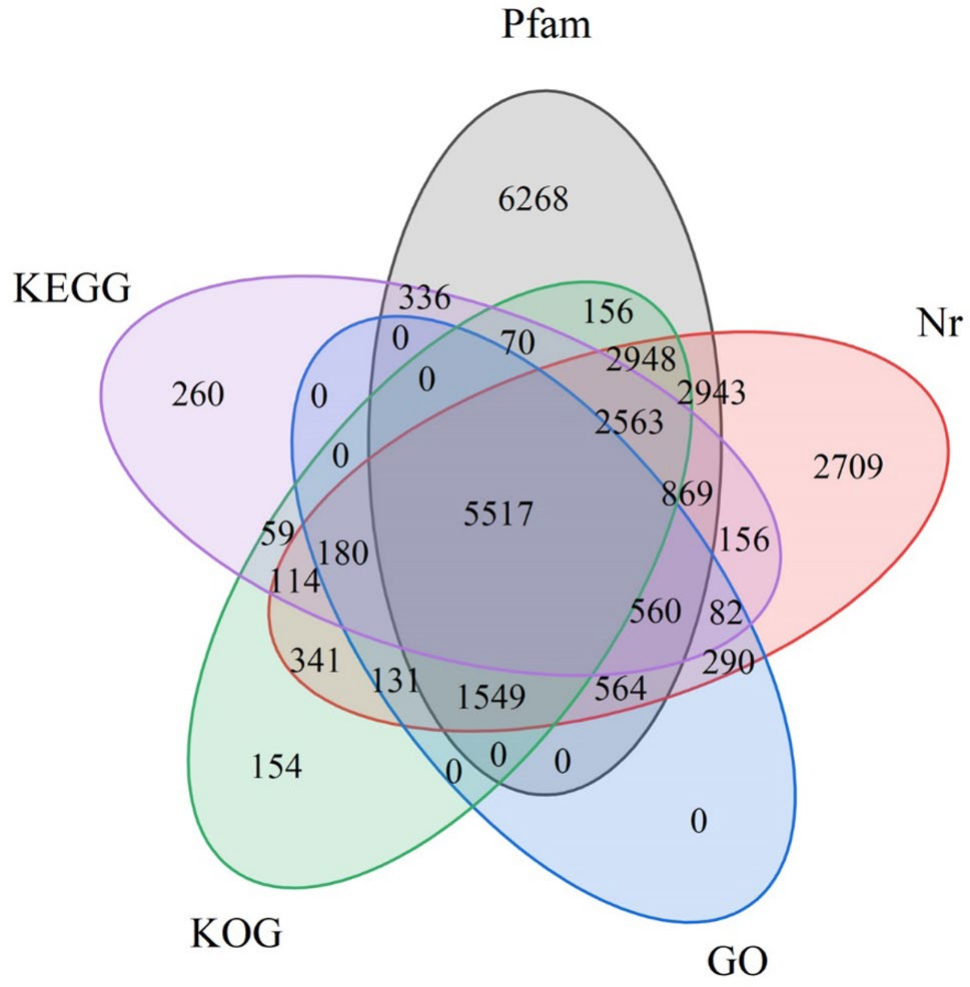
Venn diagram illustrating the numbers of unigenes expressed from public databases nr, Pfam, GO, KOG, and KEGG.

The assemblied unigenes of mantle of *P. placenta,* 113,325 in total, were further investigated by using the five public databases (Pfam, nr, GO, KOG, and KEGG) with a cutoff E-value of 10^−5^ (Fig. 2). The unigenes annotated in the Pfam database contain about 21.48% of all unigenes, followed by 18.99% annotated unigenes in the nr database, 12.16% in the KOG database, 9.5% in the KEGG database, and 7.83% in the GO database. In the alignment with NCBI nr database, 21,516 unigenes were successfully annotated (Table 3). Venn diagram of the numbers of unigenes expressed from public databases nr, Pfam, GO, KOG, and KEGG were shown in Fig. 2, which show clearly that many of the unigenes were annotated from more than one database.

**Table 3:** Statistics of the numbers and percentages of unigenes annotated in different databases and percentage of each database.

The database to be used for annotation	Number of annotated unigenes	Percentage (%) of annotated unigenes
nr	21,516	18.99
KEGG	10,766	9.5
Pfam	24,343	21.48
GO	8873	7.83
KOG	13,782	12.16

**Figure 3:**
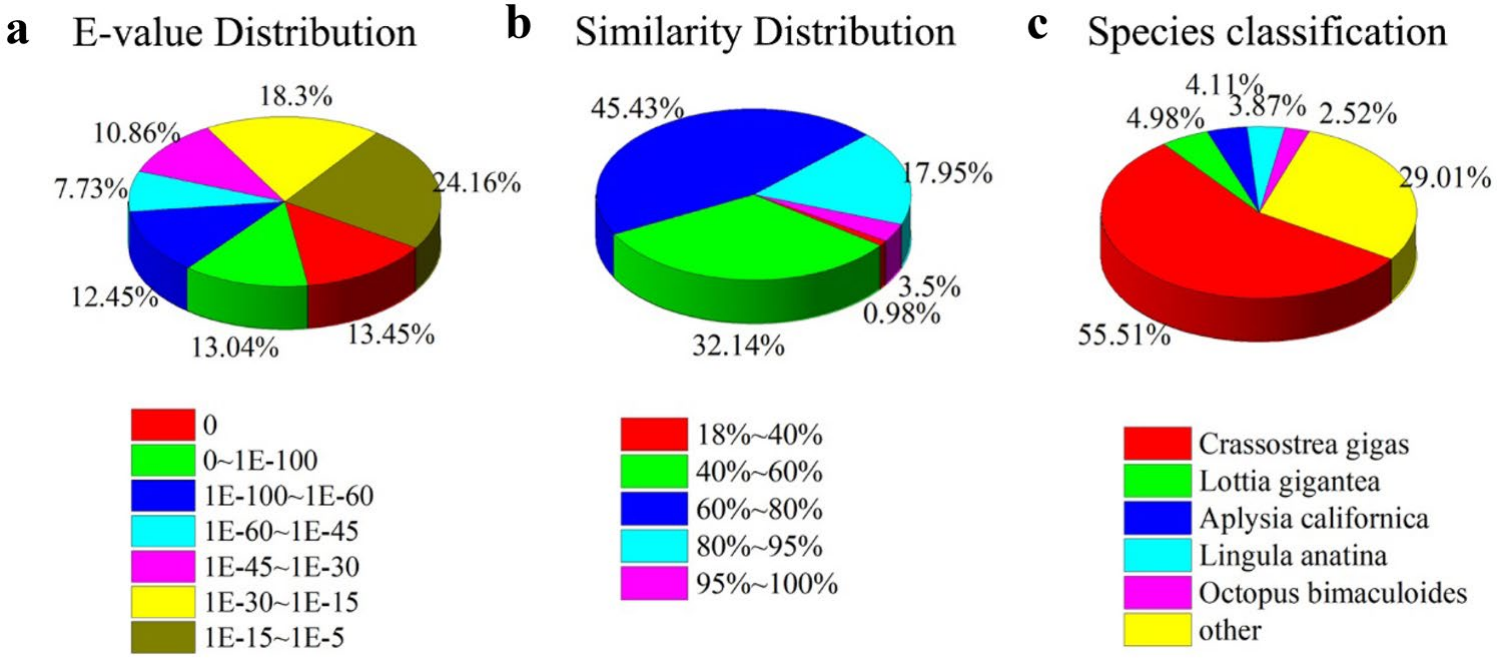
Distribution of E-values (**a**), and similarities (**b**) of annotated unigenes from the mantle tissue of *P. placenta,* and the homologous comparison of annotated unigenes from *P. placenta* and other species (**c**) according to nr database.

Among these successfully annotated unigenes, 46.67% of the annotated unigenes have strong homology with the aligned proteins (E-values less than 1.0E^-45^) in the nr database (Fig. 3a). Nearly half of the unigenes (45.43%) return 60–80% similarities to annotated unigenes, and 21.45% return 80–100% similarity to annotated unigenes (Fig. 3b). 55.51% of the annotated unigenes of mantle of *P. placenta* matches best with that extracted from pacific oyster *C. gigas* according to nr database (Fig. 3c). A possible reason for the above homologous genes of the two different animals might be that the *C. gigas* is one of the few molluscans whose genomes have been completely sequenced and the details of numerous genes of *C. gigas* have been released. The strong similarity revealed in our study further implies that the genome studies of *C. gigas* can be reliable references for the study of *P. placenta* even though the evolutionary relationship between *C. gigas* and *P. placenta* is not very close.

**Figure 4:**
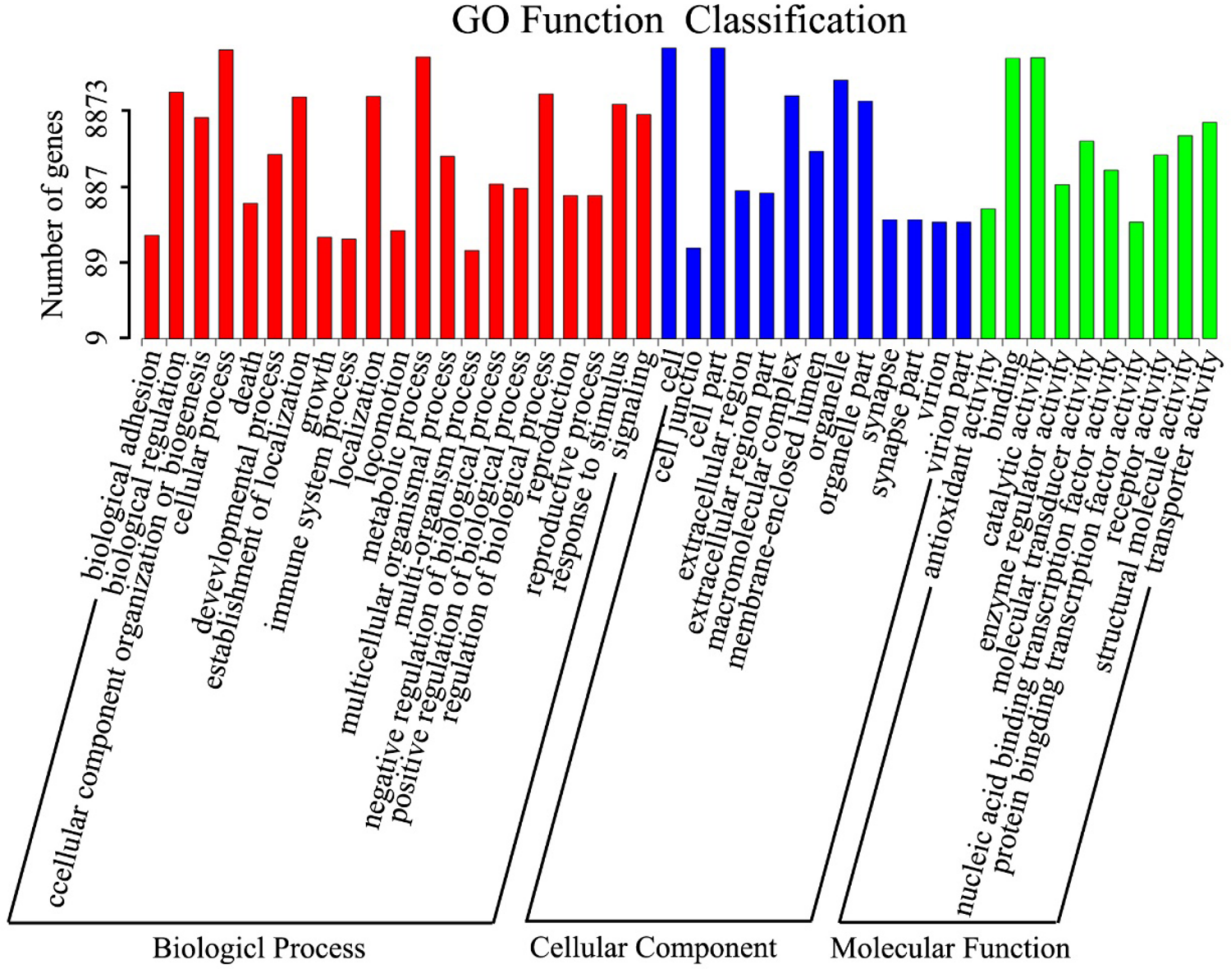
GO categorizations (Biological processes, Cellular components and Molecular functions) of unigenes in the mantle transcriptome of *P. placenta.*

To classify the gene functions, the mantle unigenes of *P. placenta* were investigated by using the GO database. This database is an international standardized gene functional classification system that offers a dynamically updated controlled vocabulary and strict definitions that completely describe the properties of genes and their products in any organism. There are 8,873 unigenes assigned to three main GO categories (Table 3), including biological process category, cellular component category, and molecular function category, and 44 terms in total as shown in Fig. 4. For GO categorization, the “metabolic process” and “cellular process” terms are dominant in the biological process category, the “cell” and “cell part” terms are dominant in the cellular component category, and the “binding” and “catalytic activity” terms were dominant in the molecular function category. The terms “extracellular region” and “extracellular matrix” associated to cellular component category are generally supposed to be related to SMPs. Most SMPs were up-regulated to a greater content at the juvenile stage than those at the adult stage of pearl oyster *P. fucata* ^50^. A total of 31 unigenes related to SMPs are recognized in the terms “extracellular region” from the mantle tissue of *P. placenta*, such as collagen, chitin binding protein, calcium ion binding protein (Excel S1).

**Figure 5:**
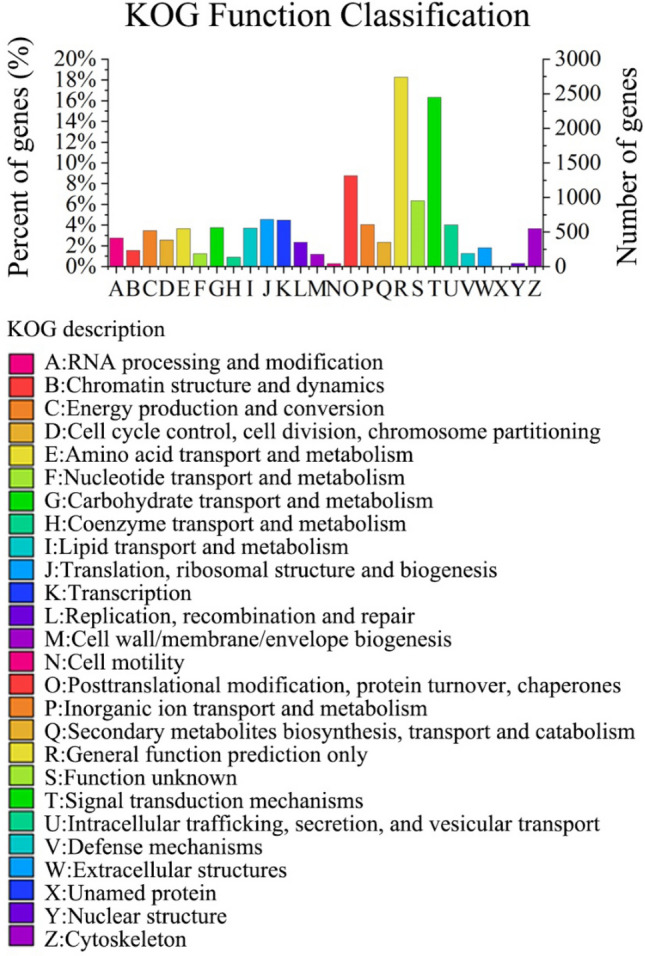
KOG categorizations of unigenes in the mantle transcriptome of *P. placenta*.

The mantle unigenes of *P. placenta* were investigated by using the KOG database for better functional classification. 13,782 unigenes of mantle tissues of *P. placenta* were assigned to 26 categories according to KOG database (Table 3). About 41% of the unigenes are assigned to the three main categories, (R) category “general function prediction only” (2739, 17.62%), (T) category “signal transduction mechanisms” (2445, 15.73%), (O) category “posttranslational modification, protein turnover, chaperones” (1314; 8.5%). In this study, the numbers of unigenes of mantle tissue *P. placenta* assigned to the P (inorganic ion transport and metabolism) category and T category were 606 (3.9%) and 2445 (15.73%), respectively (Fig. 5, Excel S2). Many of the annotated unigenes assigned to T and P categories in Excel S2 are homologous with ferritin, calcium ion binding protein, calmodulin and other biomineralization-related proteins as reported in the literature. The P category (inorganic ion transport and metabolism) contains some Ca^2+^-related proteins such as Ca^2+^ binding protein, Ca^2+^ transporting ATPase, Voltage-gated Ca^2+^ channels (Excel S2). In contrast, there are less than 10% unigenes unevenly distributed in the remaining categories such as Y category “nuclear structure” and N category “cell motility”, accounting for 0.33% and 0.3%, respectively (Fig. 5).

**Figure 6:**
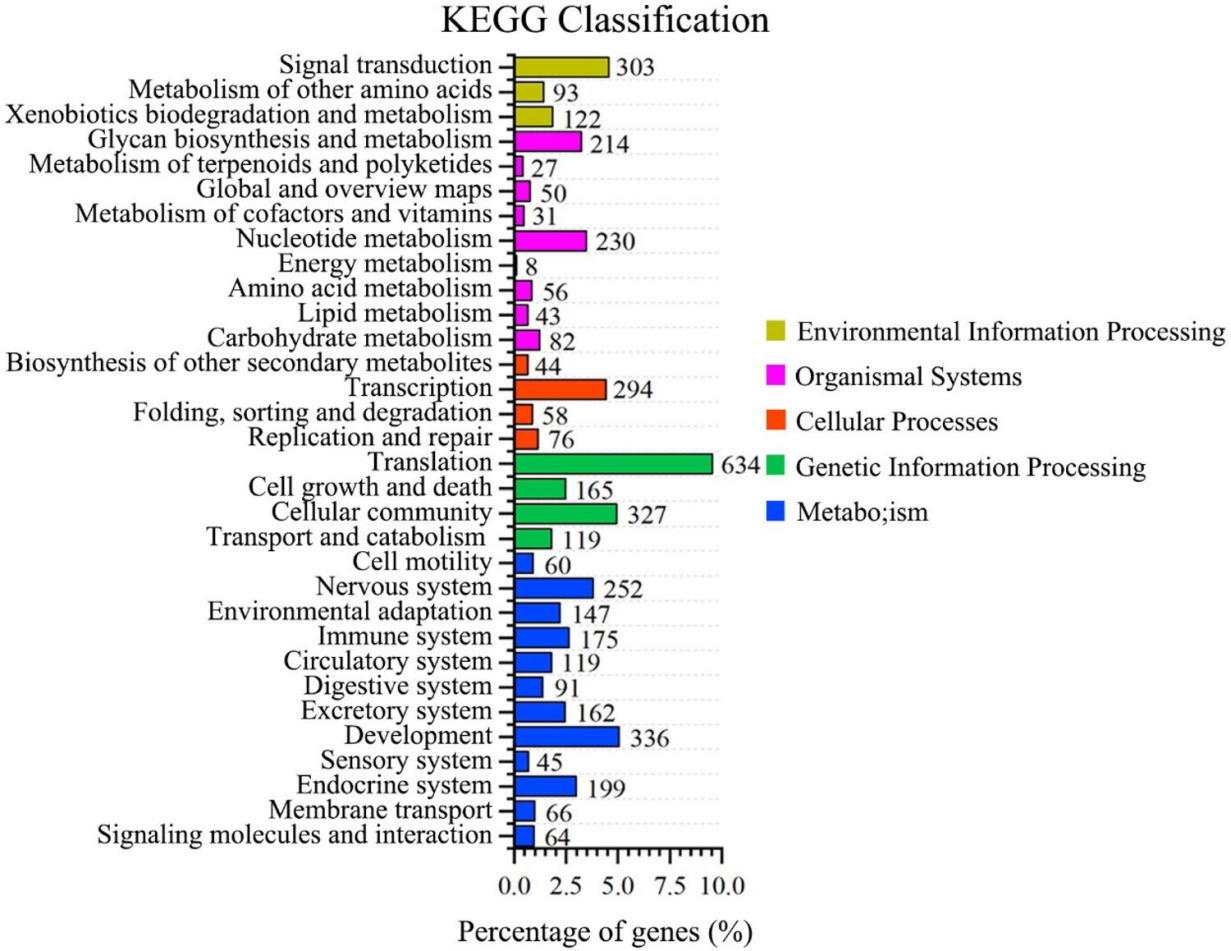
KEGG assignment of unigenes in the mantle transcriptome of *P. placenta.*

To better understand biological pathways involved in the *P. placenta* mantle unigenes, all unigenes of mantle tissues were assigned according to the reference canonical pathways in the KEGG database. According to the classification results, 10,766 unigenes were assigned to five specific pathways (Table 3). 1,716 unigenes were assigned to metabolism, followed by genetic information processing (1,245 unigenes), organismal systems (741 unigenes), environmental information processing (518 unigenes), cellular processes (472 unigenes). According to the KEGG classification, the pathway of translation owns the largest number of unigenes (634 unigenes), representing for 50.92% of the total annotated unigenes in the category of genetic information processes, followed by pathway of global and overview maps (336 unigenes) in metabolism category (Fig. 6). It was reported that the calcium signaling pathway, Wnt (wingless/int1) signaling and tyrosine metabolism pathways probably play significant roles in the biomineralization process ^37,50^. However, no unigene was found in the calcium signaling pathway in the KEGG database from the unigenes of the mantle tissue of *P. placenta*. 23 unigenes were found to be involved in Wnt signaling and tyrosine metabolism pathways. Among them, the calcyclin binding-protein, frizzled-related protein and dopamine beta-monooxygenase proteins are supposed to be related to calcium signal transduction and the shell formation process (Excel S3). The chemokine signaling and leukocyte transendothelial migration pathways were significantly enriched after the shell damage treatment, indicating that granulocytic hemocytes containing matrix proteins, a type of leukocyte, may transport calcium ions or contribute to the biomineralization process of calcite crystals ^51–53^. There are four unigenes found in these two signaling pathways according to KEGG database and only C–C motif chemokine may involve the biomineralization process (Excel S2).

### SNPs, Indels, SSR markers identification and primer design

**Figure 7:**
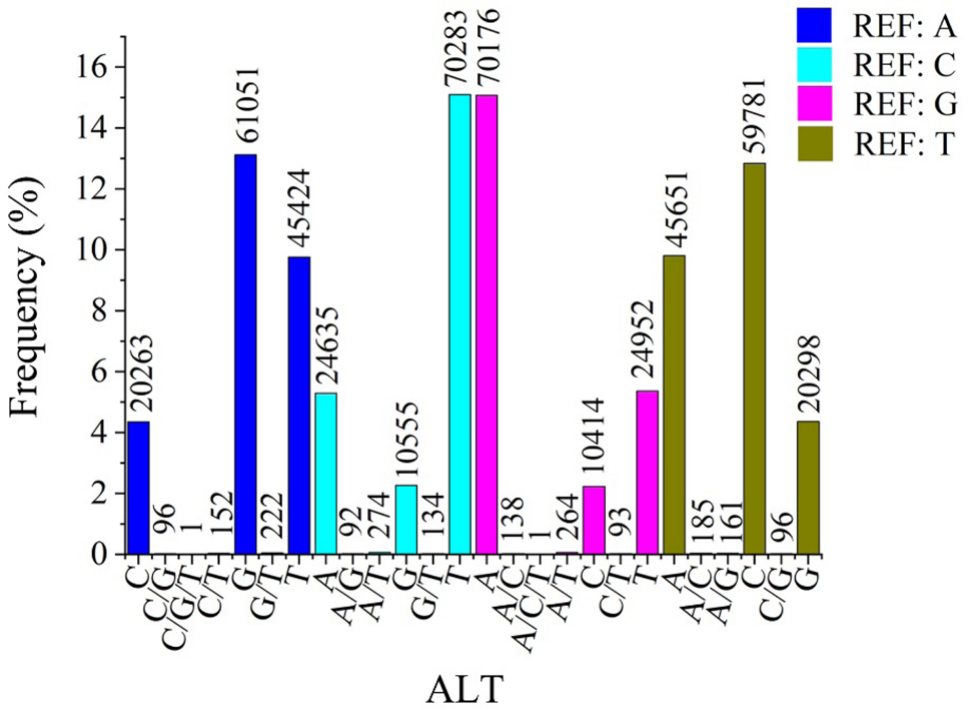
SNP types and frequencies from *P. placenta* mantle tissue. REF: The genotypes of the reference sequences at the defined sites, ALT: Other genotypes at the reference sites.

SNPs are potentially useful for genetic linkage mapping and quantitative trait locus analysis. There is no reported literature related to the SNP identification of *P. placenta*. In this study, polymorphism analysis identified 465,392 potential SNPs from 60,371 unigenes with frequency of about 7.56 SNPs per unigene (Excel S5). The numbers of transition (261,292) and transversion (202,192) mutations accounted for 56.14% and 43.45% in SNP types, respectively. The most abundant type of base variations is C/T polymorphism (70,283; 15.1%), because C in CG base is often methylated and transfers to thymine after spontaneous deamination. Another abundant type of base variations is A/T polymorphism, such as G to A (70,176; 15.08%), A to G (61,051; 13.12%) (Fig. 7). 62,103 potential indels in total were also identified among 21,630 unigenes with frequency of about 2.87 indels per unigene (Excel S6). The numbers of unigenes decrease gradually with the increase of indel length from 1 to 10 bp (Fig. S1). The indel lengths are mainly 1, 2 and 3 bp. The numbers of deletion are larger than that of insertion when the indel length is more than 10 bp (Fig. S1).

**Figure 8:**
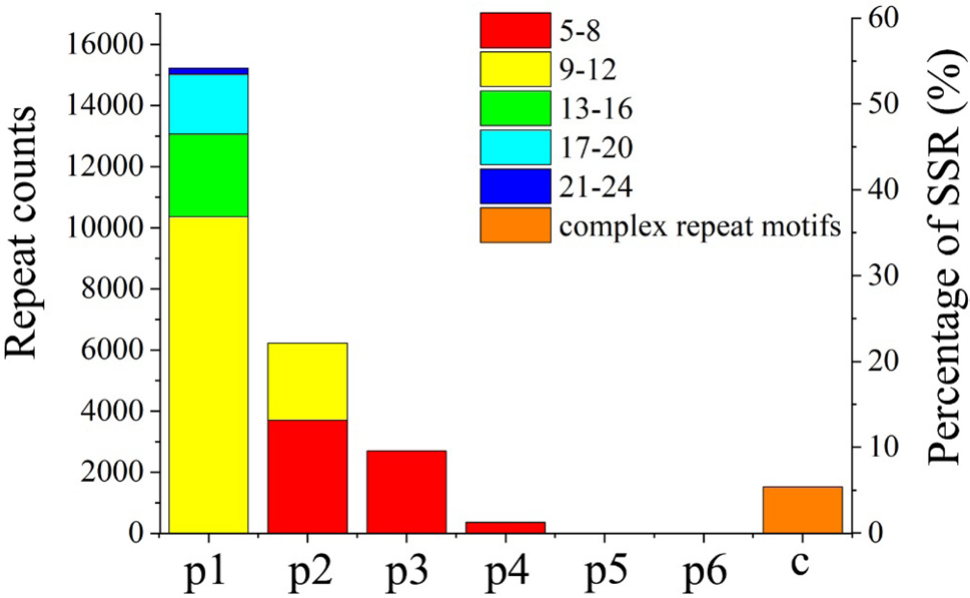
Distribution of SSRs based on the number of repeat units. p1: mono-nucleotide, p2: di-nucleotide, p3: tri-nucleotide, p4: tetra-nucleotide, p5: penta-nucleotide, p6: hexa-nucleotide, c: complex repeat motifs.

In addition, 21,640 unigenes containing 26,048 potential SSR markers were identified (Fig. 8). Among these unigenes, the most abundant type of repeats is p1 (mono-nucleotide) (15,224; 58.4%), followed by p2 (di-nucleotide) (6227; 23.9%), p3 (tri-nucleotide) (2700; 10.3%), p4 (tetra-nucleotide) (361; 1.3%) repeats. Furthermore, there are some c- types (complex repeat motifs), accounting for 5.8% (1524), different from the above mentioned nucleotides in these SSR markers. The frequencies of SSRs with different numbers of repeat units were calculated. Among the p1 repeat units, the counts of the repeat units between 9 and 12 are dominant, accounting for more than 50%, followed by 13–16 repeat units (17.74%) and 17–20 repeat units (12.85%). More than half of 5–8 repeat units were assigned to the p2 repeat units, followed by 9–12 repeat units. However, only 5–8 repeat units are present in both p3 and p4 repeats (Fig. 8). The A/T repeat units of mono-nucleotide are dominant among these types, accounting for 55.57%. AT/TA (9.42%) was dominant in the p2 repeat units, followed by TG/CA (4.94%), GA/GT (4.16%) (Fig. S2). ATG/GAT (2.2%) was dominant in the p3 repeat units, followed by ATC/TGA (1.71%), CAT/TCA (1.2%). The remaining types of motifs have more complicated types but less numbers (c-type, complex repeat motifs), accounted for 7.28% in total. To further test the SSR markers, forward and reverse primer pairs were obtained using Primer 3.0 (Excel S7).

### Identification of genes involved in biomineralization process

To better understand the proteins related to the shell formation process, the annotated unigenes of mantle tissues of *P. placenta* were compared with the sequences of proteins known to be associated with biomineralization process in molluscan shells using nr database. The annotated unigene with lowest E-value was selected as the representative unigene while several annotated unigenes were assigned to the same reference unigene. 178 homologous unigenes of 51 shell matrix proteins, such as calmodulin, perlucin, ferritin and carbonic anhydrase were found to be related to biomineralization process in the transcriptome of mantle tissue of *P. placenta* (Excel S10). This is the first time to report the potential biomineralization-related unigenes in the mantle tissue of *P. placenta*, as far as we know. A lot of researchers have focused on the identification of genes related to the shell formation of molluscan animals, and an increasing number of genes have been identified ^55–58^. 259 proteins were identified from oyster *C. gigas* shells by proteomic analysis ^56^. In comparison with the proteomic data of shell matrix of *C. gigas*, we identified a set of 158 unigenes that are probably related to shell formation, including house-keeping protein elongation factor 1a, and extracellular matrix protein collagen (Excel S9). Many of the shell-formation related proteins are enzymes such as glutathione peroxidase, hemicentin and tyrosinase, that may be involved in matrix construction or modification ^57,58^. In this study, only one enzyme tyrosinase (three unigenes) was found to be related to the shell formation process in the transcriptome of *P. placenta* (Excel S9). Twenty-one proteins with 66 homologous unigenes were identified to be related to calcitic shell formation of *P. placenta* (Table 4). Furthermore, eighteen of the above 66 unigenes were identified to be highly expressed in mantle tissue of *P. placenta.* A few highly expressed unigenes with fragments per kilobase of feature per million mapped reads (FPKM) values of > 15 are carbonic anhydrase, calreticulin, ferritin, perlucin, gigasin-2, and tyrosinase-like proteins.

**Table 4:** Identification of genes involved in the calcitic shell formation of *P. placenta*^a^*.*

Accession	Gene_name	Gene_ID	nr_Evalue	nr_Description	Low FPKM	High FPKM
EKC34657	Peroxidase1 ^56^	c84192_g1	0	Chorion peroxidase [*Crassostrea gigas*]	27.46	63.27
EKC26108	Peroxidase2 ^56^					
AAV69062.1	Alkaline phosphatase ^56,59^	c84312_g1	3.00E−156	PREDICTED: alkaline phosphatase-like [*Lingula anatina*]	6.41	13.58
		c84796_g1	1.00E−156	Alkaline phosphatase, tissue-nonspecific isozyme [*Crassostrea gigas*]	4.31	8
BAG68618.1	BMP2/4 ^60,61^	c349_g1	5.00E−31	Bone morphogenetic protein 2/4 [*Lymnaea stagnalis*]	0.26	2.13
		c123147_g1	5.00E−06	Bone morphogenetic protein 2/4 [*Septifer virgatus*]	0	10.27
BAJ52887.1	Carbonic anhydrase(CA), similar to Nacrein ^56^	c84941_g5	1.00E−45	PREDICTED: carbonic anhydrase-like [*Crassostrea gigas*]	126.8	244.22
		c85950_g1	2.00E−39	PREDICTED: carbonic anhydrase 1-like [*Crassostrea gigas*]	103.95	139.29
		c71108_g1	8.00E−77	Carbonic anhydrase 2 [*Crassostrea gigas*]	67.5	124.48
		c81423_g3	1.00E−69	PREDICTED: carbonic anhydrase 1-like [*Crassostrea gigas*]	27.26	45.21
ACI22622.1	Calmodulin ^56^	c114357_g1	8.00E−11	PREDICTED: calmodulin-like [*Crassostrea gigas*]	2.41	7.74
		c10600_g1	3.00E−09	RecName: Full = Calmodulin; Short = CaM	0.81	3.51
		c78814_g1	7.00E−29	RecName: Full = Calmodulin; Short = CaM	0.69	2.77
		c65518_g1	2.00E−12	RecName: Full = Calmodulin; Short = CaM	0.15	2.93
		c31509_g1	9.00E−28	Calmodulin [*Crassostrea gigas*]	0	1.12
		c102707_g1	2.00E−12	RecName: Full = Calmodulin; Short = CaM	0	0
		c52942_g1	4.00E−06	RecName: Full = Calmodulin-2; Short = CaM 2	0	0.38
		c60826_g1	1.00E−94	RecName: Full = Calmodulin; Short = CaM	0	1.38
		c62972_g1	8.00E−12	Calmodulin [*Crassostrea gigas*]	0	0
		c105690_g1	1.00E−08	PREDICTED: calmodulin-like [*Crassostrea gigas*]	0	0
ABR68546.1	Calreticulin ^62,63^	c84621_g1	2.00E−135	Ccalreticulin-like protein [*Littorina littorea*]	114.56	172.26
		c112169_g1	6.00E−06	PREDICTED: calreticulin-like [*Biomphalaria glabrata*]	0	0
BAF73720.1	Chitin synthase/PfCHS1 ^56,64^	c86966_g4	3.00E−164	Chitin synthase, partial [*Macandrevia cranium*]	5.4	19.33
		c24142_g1	4.00E−08	Chitin synthase 1 [*Crassostrea gigas*]	0	52.05
P86734.1	Ependymin related protein 1(EDPR) ^56,59,65^	c104043_g1	3.00E−06	Mammalian ependymin-related protein 1-like [*Lingula anatina*]	0	0
		c107140_g1	1.00E−18	Mammalian ependymin-related protein 1-like [*Crassostrea gigas*]	0	0
		c110802_g1	4.00E−06	Mammalian ependymin-related protein 1-like [*Biomphalaria glabrata*]	0	3.51
AAQ12076.1	Ferritin ^66–68^	c71166_g1	2.00E−105	Ferritin [*Haliotis diversicolor supertexta*]	2598	5972.88
		c54191_g1	2.00E−41	Ferritin [*Scapharca broughtonii*]	0	0
		c90808_g1	3.00E−17	Ferritin [*Conus novaehollandiae*]	0	0
P86785	Gigasin-2 ^56^	c76266_g1	5.00E−10	PREDICTED: gigasin-2 isoform X1 [*Crassostrea gigas*]	61.13	124.78
ADD16957.1	Perlucin ^56,69^	c67461_g2	3.00E−23	Perlucin [*Haliotis diversicolor*]	65.55	100.36
		c74134_g1	4.00E−06	Perlucin [*Hyriopsis cumingii*]	4.71	8.62
		c100512_g1	4.00E−18	Perlucin [*Haliotis diversicolor*]	0.71	1.2
		c66124_g1	3.00E−29	Perlucin [*Crassostrea gigas*]	0.28	1.45
		c15372_g1	6.00E−16	Pperlucin [*Hyriopsis cumingii*]	0	0
		c71177_g1	1.00E−08	Perlucin [*Crassostrea gigas*]	0	2.11
BAH97338.1	Protein PIF ^56^	c71325_g1	4.00E−20	PREDICTED: protein PIF-like [*Crassostrea gigas*]	31.79	44.89
		c66761_g1	5.00E−11	PREDICTED: protein PIF-like [*Crassostrea gigas*]	27.22	37.84
		c64812_g1	2.00E−06	PREDICTED: protein PIF-like [*Crassostrea gigas*]	0.45	1.42
BAA89420.1	Sarcoplasmic calcium-binding protein ^70,71^	c102115_g1	1.00E−11	Sarcoplasmic calcium-binding protein [*Meretrix lusoria*]	0	0
		c107796_g1	4.00E−30	Sarcoplasmic calcium binding protein [*Ruditapes philippinarum*]	0	1.13
AAZ66340.1	Tyrosinase-like ^56,72^	c73086_g1	1.00E−82	PREDICTED: tyrosinase-like protein [*Crassostrea gigas*]	303.51	430.78
		c73399_g1	6.00E−88	PREDICTED: tyrosinase-like protein 1 isoform X2 [*Crassostrea gigas*]	147.25	202.78
		c73399_g1	6.00E−88	PREDICTED: tyrosinase-like protein 1 isoform X2 [*Crassostrea gigas*]	147.25	202.78
		c84724_g1	1.00E−60	Tyrosinase [*Mizuhopecten yessoensis*]	4.59	15.55
		c84724_g1	1.00E−60	Tyrosinase [*Mizuhopecten yessoensis*]	4.59	15.55
		c74294_g1	6.00E−42	Putative tyrosinase-like protein tyr-3 [*Crassostrea gigas*]	4.47	9.96
		c111859_g1	2.00E−27	PREDICTED: tyrosinase-like protein [*Crassostrea gigas*]	0	0.62
		c63653_g1	3.00E−08	Putative tyrosinase-like protein tyr-3 [*Crassostrea gigas*]	0	3.21
		c6923_g2	7.00E−13	Putative tyrosinase-like protein tyr-3 [*Crassostrea gigas*]	0	0.24
		c94853_g1	3.00E−25	Tyrosinase A2 [*Pinctada maxima*]	0	2.86
P86987	Insoluble matrix shell proteins 6(IMSP6) ^56,73^	c59878_g1	1.00E−08	Insoluble matrix shell protein 6-like, partial [*Crassostrea gigas*]	41.5	55.56
		c69385_g1	8.00E−08	PREDICTED: insoluble matrix shell protein 6-like [*Crassostrea gigas*]	32.11	104.85
		c85881_g6	9.00E−11	Putative insoluble matrix shell protein 5-like protein [*Pinctada fucata*]	0.44	5.13
EKC41461	Tyrosine-protein phosphatase Lar ^56^	c85401_g1	0	Tyrosine-protein phosphatase Lar-like isoform X1 [*Crassostrea gigas*]	8.91	37.66
		c56636_g1	1.00E−05	Tyrosine-protein phosphatase Lar [*Crassostrea gigas*]	0	0
EKC31553	Amine oxidase ^56,74,75^	c85095_g1	9.00E−27	Putative amine oxidase [copper-containing] [*Biomphalaria glabrata*]	2.9	4.99
		c45366_g1	3.00E−48	Putative amine oxidase [copper-containing] [*Biomphalaria glabrata*]	1.42	5.44
		c83183_g2	1.00E−65	Putative amine oxidase [copper-containing] [*Crassostrea gigas*]	0.68	3.06
		c19032_g1	4.00E−53	Pputative amine oxidase [copper-containing] [*Biomphalaria glabrata*]	0.66	1.43
		c61900_g1	6.00E−16	Putative amine oxidase [copper-containing] [*Biomphalaria glabrata*]	0.36	2.72
		c33247_g1	1.00E−44	Putative amine oxidase [copper-containing] [*Crassostrea gigas*]	0.35	0.55
		c73931_g1	3.00E−10	Putative amine oxidase [copper-containing] [*Aplysia californica*]	0	2.02
CGI_10024867	Chitotriosidase1 ^59,75^	c51351_g1	8.00E−146	PREDICTED: chitotriosidase-1-like [*Lingula anatina*]	44.12	51.43
		c79603_g1	6.00E−125	PREDICTED: chitotriosidase-1-like, partial [*Aplysia californica*]	0.33	1.73
H2A0L6	Beta-hexosaminidase ^56^	c75081_g1	4.00E−46	RecName: Full = Putative beta-hexosaminidase; AltName: Full = Beta-N-acetylhexosaminidase [*Crassostrea gigas*]	35.32	73.48
K1QBN6	Caltractin ^76^	c70548_g1	1E−48	PREDICTED: caltractin-like [*Acropora digitifera*]	13.11	24.88
K1RCP3	Teneurin-2^56^	c59513_g1	4.00E−06	Teneurin-2 [*Crassostrea gigas*]	324.17	489.75

^a^The first column is the accession number of the protein, which could be indexed in the Uniprot. Expression level is shown as FPKM. RecName is the protein name recommended by the UniProt database. AltName represents synonyms found in the literature or in other databases.

### Comparison of the mantle transcriptomes of different molluscans

**Figure 9:**
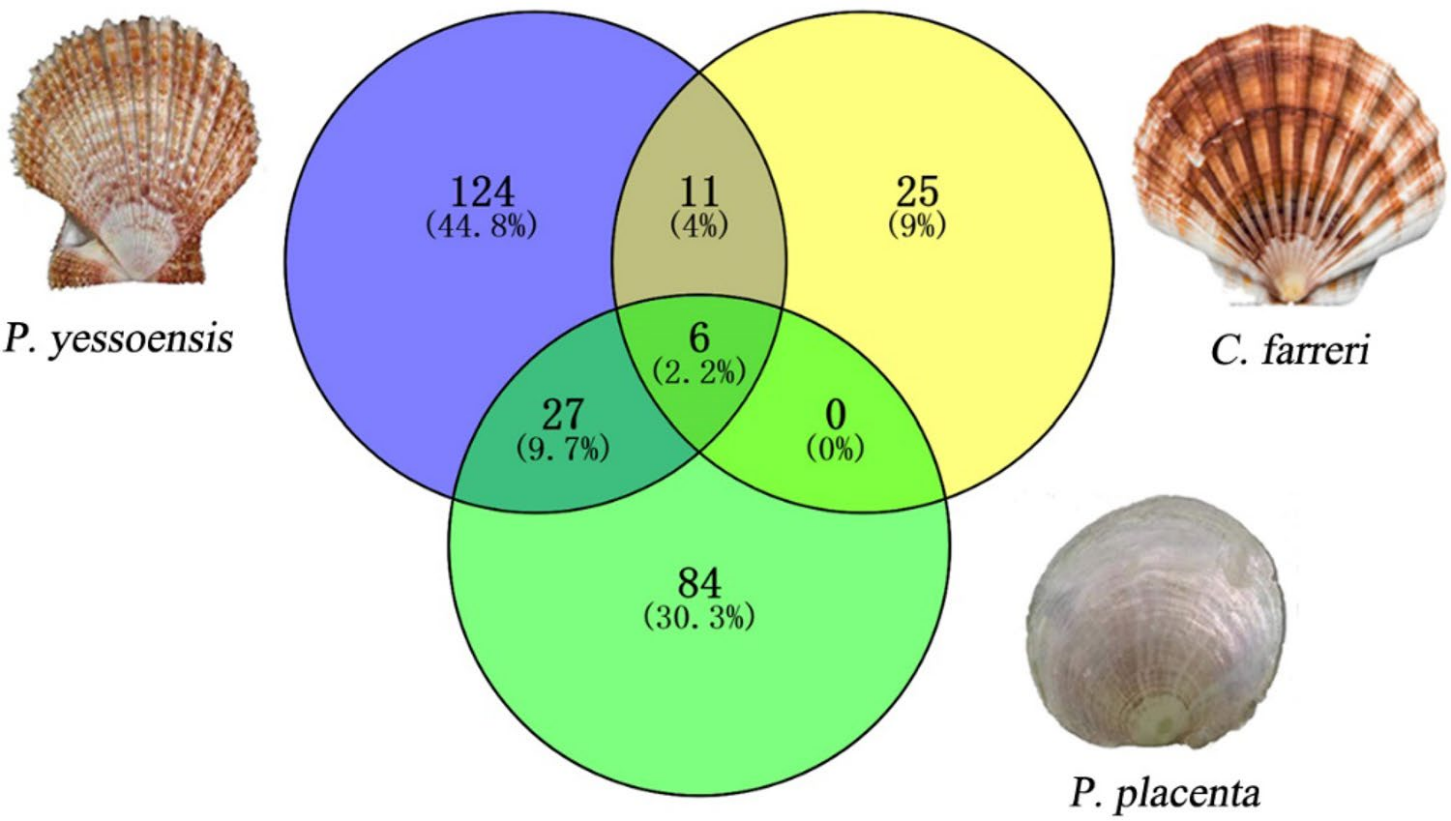
Comparison of mantle transcriptomes of three scallop shells composed of folicated calcite crystals. Blue circle: 168 biomineralization-related unigenes (124 exclusive) discovered from *P. yessoensis* mantle ^36^. Yellow circle: 42 biomineralization-related unigenes (25 exclusive) discovered from *C. farreri* mantle ^37^. Green circle: 117 biomineralization-related unigenes (84 exclusive) discovered from *P. placenta* mantle.

The shells of scallops *P. yessoensis, C. farreri and P. placenta* are composed of foliated calcite minerals. The mantle transcriptomes of scallop *P. yessoensis*, *C. farreri* and *P. placenta* were compared to find the similarities and differences of the biomineralization related proteins of these molluscan organisms ^36,37^. We find 117 biomineralization-related unigenes in the mantle of *P. placenta*, much less than those in *P. yessoensis* (162 unigenes), but much more than those in *C. farreri* (42 unigenes) (Fig. 9). There are six biomineralization-related unigenes expressed in the mantles of the three species, including sarcoplasmic calcium-binding protein, calcineurin a, calmodulin-like protein, perlucin, alkaline phosphatase, tyrosinase-like protein tyr-3. In comparison to *C. farreri, P. placenta* and *P. yessoensis* have more similar homologous biomineralization-related unigenes, 31 unigenes in total, about 27% of the biomineralization related unigenes of *P. placenta*. For example, collagen, chitin synthase 1/2, carbonic anhydrase-like, heat shock protein 70 and calmodulin were found in both *P. yessoensis* and *P. placenta*, potentially indicating their functional similarities for their biomineralization processes. There are probably more mineralization proteins to be discovered in the unigenes of the mantle tissue of *P. placenta* (Fig. 9, Excel S10).

### Quantitative Real-Time PCR (qRT-PCR) analysis

**Figure 10:**
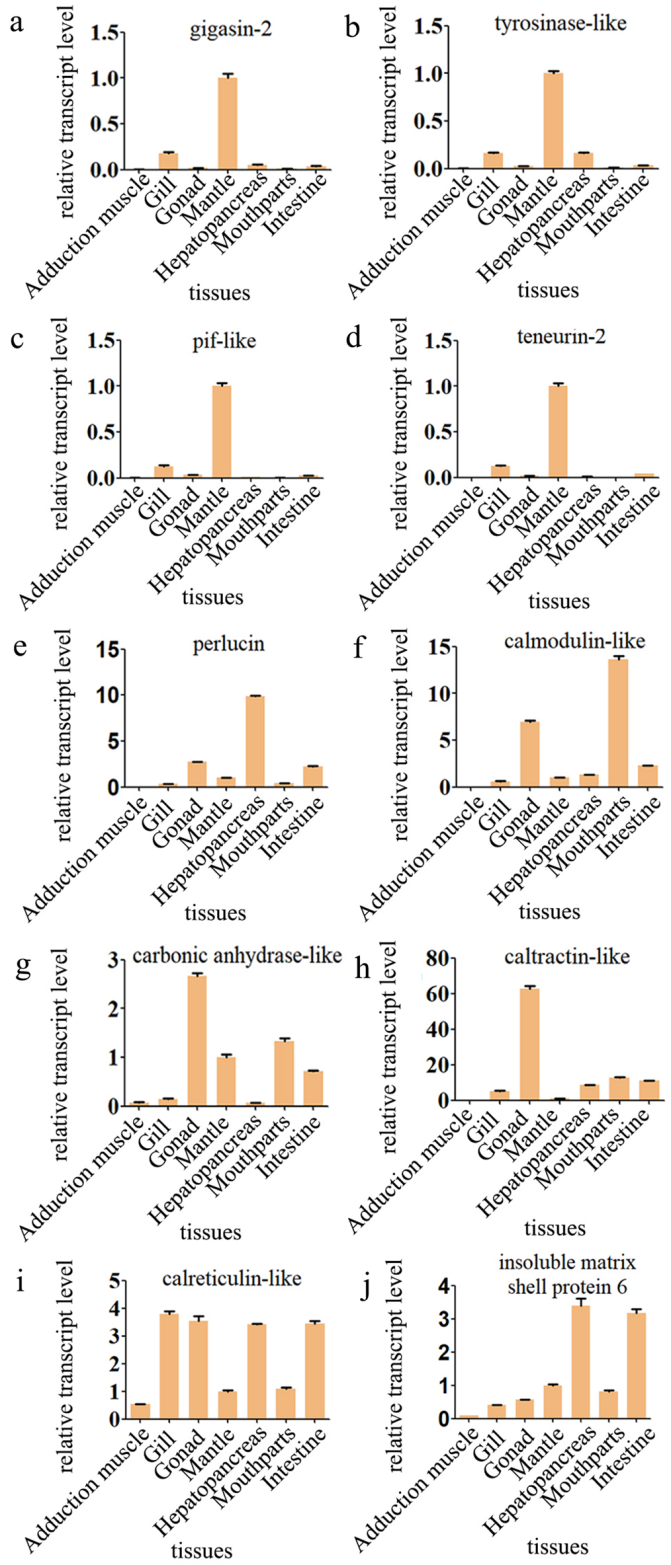
Differential expression of gigasin-2 (c76266_g1) (**a**), tyrosinase-like (c73086_g1) (**b**), pif-like(c66761_g1) (**c**), teneurin-2 (c59513_g1) (**d**), perlucin (c67461_g2) (**e**), calmodulin-like, (c81494_g1) (f), carbonic anhydrase-like (c84941_g5) (**g**), caltractin-like (c70548_g1) (**h**), calreticulin-like (c84621_g1) (**i**), and insoluble matrix shell protein 6 (c69385_g1) (**j**) in adduction muscle, gonad, gill, mantle, hepatopancreas, mouthparts, and intestine tissues of *P. placenta*, were determined using real-time PCR, The error bars represent the standard error of three biological replicates, statistical significance was considered at p < 0.05.

Ten selected potential biomineralization-related unigenes in the different tissues such as adduction muscle, gonad, gill, mantle, hepatopancreas, mouthparts, and intestine of *P. placenta* were examined by qRT-PCR (Fig. 10). In general, four of ten unigenes have much higher expression in mantle tissue than those in the other tissues, which include c76266_g1 (gigasin-2), c73086_g1 (tyrosinase-like), c66761_g1 (pif-like) and c59513_g1 (teneurin-2) unigenes (Fig. 10a–d). Pif is an important macromolecule for in vivo shell formation of nacre ^44^. In addition, it was found that pif can induce the formation of aragonite and vaterite crystals in the in vitro system ^77^. Tyrosinases are abundant in shells and their high expression in mantle of the pacific oyster *C. gigas* indicates that their functions are probably related to shell formation ^56^. Gigasin-2 and teneurin-2 were identified for the first time from the shell of *C. gigas* via shell matrix proteome characterization ^78,79^. According to the Pfam database analysis, teneurin-2 in *P. placenta* is predicted to be epidermal growth factor (EGF) domain and gigasin-2 has zona pellucida (ZP) domain (Excel S11). EGF domains are mostly found in the SMPs as tandem repeats and only present in the prismatic (calcitic) layers but not in the nacreous layer of *Pinctada*^80^. The EGF domain is a calcium-binding motif composed of 45 amino acids arranged in two small $\upbeta$-sheets with six conserved cysteine residues ^81^. Both EGF-like and ZP domains have been reported in the shells of *Lottia gigantea* ^82^ and *C. gigas* ^78^. ZP domains are present in a range of extracellular filament or matrix proteins from a wide variety of eukaryotic organisms, and are characterized by eight conserved cysteine residues, which are involved in protein polymerization processes ^83^. We propose that the above mentioned four unigenes are primarily biomineralization-related unigenes. However, the expression levels of the other six unigenes in the mantle tissue are not as high as their expression levels in the other tissues, indicating that they probably have other functions except for the biomineralization process (Fig. 10e–j). We would discuss more about the functions of these ten unigenes in the discussion part.

### Pfam database analysis

Pfam database search is important for understanding the possible biomineralization-related functions of the shell matrix proteins of molluscan animals. In molluscan animals, shell matrix proteins are very often repetitive, highly conserved, low complex domains. The functions of these protein domains involved in biomineralization have been studied by many groups ^13,84^. For example, the nacre protein perlucin contains a C-type lectin domain and has a broad carbohydrate-binding feature, which was supposed to facilitate calcium-dependent glycoprotein-protein interactions within the skeletal matrix ^23^. In molluscan animals, the pif-like proteins contain von willebrand factor type A (VWA), chitin-binding and laminin G domains, which can bind chitin framework and accelerate CaCO_3_ precipitation inside the chitin membrane, and then regulate their vertical alignment ^85,86^. In bivalves, most EF-hand proteins from the mantle tissue of bivalves are Ca^2+^ sensors or signal modulators, which may induce conformational change by binding with Ca^2+^, such as calmodulin, troponin C and myosin light chains ^87^.

In this study, the sequences of potential biomineralization genes were obtained from the transcriptomics analysis of *P. placenta* mantle. Based on these amino acid sequences, the information of their domain was obtained by using Pfam. Finally, we speculate on the function of potential biomineralization-related proteins of *P. placenta* based on the characteristics of the domain found in biomineralization proteins of molluscan. Identification of the possible biomineralization-related functions of the unigenes expressed in the mantle tissue of *P. placenta* were carried out by keyword searching according to the domain which were reported to be related to biomineralization in the Pfam database. Many potential biomineralization-related unigenes are proposed to be involved in the shell formation from the transcriptomes of the mantle tissue of *P. placenta* according to the Pfam database analysis. (Excel S11).

Calmodulin, calponin and mucin proteins are supposed to be associated with molluscan shell formation. Among those proteins, calponin was highly expressed in the mantle of *P. placenta*, with FPKM values > 551, but the expression values of most of the other two proteins are relatively low. Only c81494_g1 unigene (calmodulin-like protein) had a high expression level (FPKM values in between 38.17 and 76.44) ( Excel S11), but it expressed a higher quantity in mouth parts than that in the other tissues (Fig. 10f), suggesting that calmodulin may play other roles in *P. placenta*. The insoluble matrix shell protein 6 shows a higher expression in the tissues of hepatopancreas and intestine than that of the other tissues in *P. placenta* (Fig. 10j).

Cadherin and collagen proteins contain enriched amount of von willebrand factor type A and epidermal growth factor domains, indicating that they were derived from the extracellular matrix ^88^. However, most of them show a low expression level in the mantle tissue of *P. placenta*. Only c83310_g1 (collagen alpha-1) shows a relatively high expression level (FPKM values in between 11.18 and 32.32) (Excel S11). Perlucin extracted from abalone nacre contains a functional C-type lectin domain which can increase the precipitation rate of calcium carbonate from a saturated solution, indicating that it may promote the nucleation and/or the growth of calcium carbonate crystals ^15,23^. Among the proteins containing perlucin domains, only the c67461_g2 unigene (perlucin) shows a high expression level (FPKM values: 65.55–100.36) in the mantle tissue of *P. placenta* (Excel S11). However, c67461_g2 unigene shows higher expression in tissues of hepatopancreas than that of the other tissues by qRT-PCR (Fig. 10e). Carbonic_anhydrase domain was reported to be involved in the formation of both nacreous and prismatic layers ^20,24,89^. The expression values of all the carbonic anhydrase unigenes are relatively high, with FPKM values $>$ 27, such as c71108_g1, c85950_g1, c81423_g3 and c84941_g5 unigenes (Excel S11). At the mean time, the expression of c84941_g5 was higher in gill than that in the other tissues (Fig. 10g). On the other hand, some extracellular enzymes or inorganic ion-binding proteins such as chitinase, hemicentin and peroxidasin with relatively high expression levels in the mantle tissue of *P. placenta* are probably involved in the shell formation (Excel S11). Chitinase in shell matrix may reconstruct the chitinaceous scaffold and promote the interaction between chitin and chitin binding protein ^82^. In comparison to the other ion-binding proteins, the c71155_g1 unigene (chitinase 1) has a relatively high expression level (FPKM values in between 96.85 and 125.15) (Excel S11), but we didn't know yet how specific this gene is in each tissue of *P. placenta*.

## Discussion

Shell formation is a very complicated process that involves a series of proteins and genes, while living organisms produce biominerals with superior mechanical properties under biological control ^1^. The main objective of this study is to identify unigenes involved in biomineralization. Our study indicates that a high-coverage expression profile can be produced by using short-read Illumina sequencing technology and some effective sequence assembly tools such as Trinity and ESTScan softwares. A total of 113,325 unigenes with an average length of 697 bp was generated from the m antle tissue of *P. placenta* by using Illumina HiSeqTM 4000 sequencing technology, while 66.39% of the above unigenes (76,237) have lengths less than 500 bp (Fig. 1). The lengths of the unigene sequences from the mantle tissue of *P. placenta* are larger than those generated in the reported unigene sequences of *P. penguin* ^34^. These unigene sequences are similar to the reports for the yesso scallop *P. yessoensis* (93,204 unigenes; 733 bp) ^36^ and the freshwater pearl mussel *C. plicata* (98,501 unigenes; 689 bp) ^33^. However, the number and mean length of the unigenes from the mantle tissue of *P. placenta* are larger than that from the zhikong scallop *C. farreri* (77,975 unigenes; 538 bp) ^37^ and the pearl oyster *P. maxima* (108,704 unigenes; 407 bp) ^90^. This difference may be attributed to the use of different sequencing platforms. A large quantity of genomic data is available for many bivalve species, but only 18.99% of the mantle tissue of *P. placenta* were annotated in nr database. This means that more than half of the unigenes of the mantle tissue of *P. placenta* have no known homologous unigenes. The low rate of annotated unigenes of the mantle tissue of *P. placenta* could be a result of limitations in the genomic information available for *P. placenta*, which is the case in many of other bivalve species ^33,36^.

The SNPs of the mantle tissue of *P. placenta* were obtained from the sequencing errors as well as the true SNPs. In our data, the obtained SNP density was 0.0059 SNPs/bp (0.59%) in the mantle tissue of *P. placenta* (Excel S5, Table 2), which was significantly larger than the sequencing error (0.01%) (Table 1). This result confirmed the reliability of the obtained SNPs data. The SNPs are potentially useful for genetic linkage mapping and for the analysis of quantitative traits of the *P. placenta*. The transcriptome we present here provides the most comprehensive polymorphism for the *P. placenta* to date, as far as we know. The SNP density of the eastern oyster *C. virginica* is 0.042 SNPs/bp ^54^, which was more polymorphic than that of the mantle of *P. placenta* (0.0059 SNPs/bp) (Excel S5, Table 2). 465,392 potential SNPs were constructed from 60,371 unigenes, with frequency of about 7.56 SNPs per unigene in the mantle tissue of *P. placenta*, which was consistent with the reported result of the mantle tissue of pearl oyster *P. martensii* ^35^. The indel density of the mantle tissue of *P. placenta* was 2.87 indels per unigene (Excel S6), which was much lower than that indel density of the mantle tissue of *P. martensii* ^35^. As is well known, the Illumina sequencing is considered to be robust against homopolymer errors and therefore it may be suited well for identification of indels ^91^.

The variations in unigene expression between different tissues have been shown to be correlated with shell formation in molluscans such as *C. gigas* ^22^, *P. penguin* ^34^, and *T. pyramis* ^32^. The exact biomineralization functions of proteins such as perlucin ^15^ and pif ^85^ have been investigated by in vitro and in vivo mineralization studies. According to the nr database, 178 potential biomineralization-related unigenes were identified in the mantle transcriptome of *P. placenta* in this work. Among these unigenes*,* ten selected potential biomineralization-related unigenes (FPKM values $>$ 15) were examined in the different tissues of *P. placenta* by qRT-PCR. In the present study, four of ten unigenes (gigasin-2, tyrosinase-like, pif-like and teneurin-2) have much higher expression in the mantle tissue than those in the other tissues, indicating that they are very often related with the biomineralization process of *P. placenta* shell (Fig. 10a–d). Three gigasin-2 isoforms were identified in water soluble matrix of *C. gigas* shell, which are proposed to be involved in bone remodeling processes and could be responsible for the biocompatibility between bone and nacre grafts ^79^. Meanwhile, gigasin-2 was highly expressed in the mantle tissue of *C. gigas* ^56^. However, homologous proteins have not been identified in other species since gigasin-2 was reported in 2012 ^56^. It is known that tyrosinase family are potentially involved in melanin biosynthetic pathway in various organisms. Moreover, it was reported that tyrosinases from molluscans are secreted from the mantle and transported to the prismatic layer of the shell, while they contribute to melanin biosynthesis and shell pigmentation ^56,92,93^. In this study, there is no pigmentation in the mantle tissue and the transparent shell of *P. placenta*. Therefore, the high expression of tyrosinase in the mantle tissue indicates that their functions are not only related to melanin biosynthesis, but also related to the shell formation. After injection of pif dsRNA, both of calcite laths of the *C. cigas* shell and nacreous layer of the *P. fucata* shell grew to disordered structure in vivo, indicating that pif protein might be essential for the normal growth of the prismatic and nacreous layer ^44,77^. The teneurin-2 was first identified from diverse shell matrix proteome and had signal peptides in *C. gigas*, and it was proposed that it was secreted from the mantle into the shell ^78^. According to the above discussion about the expressions of the four unigenes and their functions for biomineralization process in the other molluscan species in the literatures, we propose that gigasin-2, tyrosinase-like, pif-like and teneurin-2 may play important roles for the biomineralization process. It would be important to extract these four proteins and investigate their functions for biomineralization via in-vitro crystallization process of CaCO_3_ in the future. We consider to study the full-length cDNA sequences, gene expression and recombinant proteins of these four unigenes to understand their functions for in vivo and in vitro crystallization of CaCO_3_.

Six of the remaining unigenes don’t exhibit high expression in the mantle tissue, in comparison to the other tissues of *P. placenta*. Thus, it is hard to tell whether they participate in biomineralization or not based on the qRT-PCR analysis. These findings are somewhat similar from those of previous studies. For example, the researchers identified six types of perlucin and discovered their different expression levels in different tissues of *T. pyramis*. Some of the perlucin proteins were expressed at the highest levels in the digestive gland, while the others were expressed at high levels in the mantle or the gonad of *T. pyramis* ^32^. The perlucin was isolated from the nacreous layer of the marine snail *Haliotis laevigata* and it could promote the nucleation of CaCO_3_ crystals on the calcite surface in the in vitro experiments ^94^. In addition, calmodulin-like protein can induce the nucleation of aragonite through binding with the 16-kDa protein and regulates the growth of calcite in the prismatic layer of pearl oyster *P. fucata* ^95^. This expression pattern and the in vitro crystallization experiments suggest that perlucin family may play important roles in both of the biomineralization process and digestive process ^32,94^. Similar to the perlucin, two types of calmodulin were expressed at the lowest level in the mantle than the other tissues in *T. pyramis* ^32^. Calmodulin-like protein was expressed with the highest level in the mantle tissue of *P. fucata* species and has a potentially high affinity for calcium ^96^. The carbonic anhydrase family were expressed in the mantle and associated with the shell formation in the european abalone *Haliotis tuberculata* ^97^. Nacrein containing carbonic anhydrase domain was expressed in both the nacreous layer and the prismatic layer of *P. fucata* ^24^. Meanwhile, it was also highly expressed in the mantle of *T. pyramis* and *P. penguin* ^32,34^. However, the researchers analyzed the expression levels of the five studied carbonic anhydrase isoforms in different tissues and found that four of them were more highly expressed in the hemocytes than in the gills or the mantle in *C. gigas* ^22^. In *P. placenta*, one of the perlucin family was identified by qRT-PCR and showed a highest expression in the digestive gland than that in the other tissues (Fig. 10e). The expression level of c81494_g1 unnigene (calmodulin-like) was the highest in the mouthpart, moderate in the gills, very low in the mantle tissue (Fig. 10f). The expression of carbonic anhydrase-like was the highest level in the gill tissue, medium in the mantle in *P. placenta* (Fig. 10g). Based on the qRT-PCR and in vitro crystallization results in the reported literature, we conclude that the above mentioned three unigenes, perlucin, calmodulin-like and carbonic anhydrate-like unigenes are potentially related to the biomineralization process of *P. placenta.*

As a member of the calmodulin subfamily of EF-hand Ca^2+^-binding proteins, Caltractin was first identified in *C. gigas* ^56,98^. Another unigene calreticulin is also a calcium-binding protein, it was primarily involved in the unfolded protein response to cellular stress (temperature, salinity, air exposure and heavy metals) in the endoplasmic reticulum ^14,56,99^. Both of the two calcium-binding proteins, calreticulin-like and caltractin-like exhibit relatively low expression in the mantle tissue according to the qRT-PCR results (Fig. 10h,i). However, we consider that they may probably have some kind of function for biomineralization process since they are calcium-binding proteins ^100–102^.

## Conclusions

In conclusion, the transcript dataset of the mantle tissue of *P. placenta* was investigated in details by using Illumina HiSeqTM 4000 platform and public unigene databases. The identified and annotated unigenes provide valuable genomic resources for the understanding of the biomineralization mechanism. More than half of the annotated unigenes of the mantle tissue of *P. placenta* are consistent with those proteins from the pacific oyster *C. gigas* according to nr database. The transcripts of mantle tissue of *P. placenta* were identified with SNP, SSR and indel markers. These SNP markers, SSR markers and primers may be used in the construction of a genetic linkage map and gene-based association studies. 66 homologous unigenes of 21 shell matrix proteins in the transcriptome of mantle tissue of *P. placenta* were found to be related to the calcitic shell formation, while eighteen of the above unigenes are highly expressed with FPKM larger than 15 in the mantle tissue. Furthermore, qRT-PCR analysis for ten of highly expressed homologous unigenes (FPKM > 50) related to biomineralization from six different tissues of *P. placenta* indicate that seven of them are potentially related to the biomineralization process of the calcitic shells of *P. placenta*. Especially, the qRT-PCR analysis shows that four of ten examined unigenes including teneurin-2, gigasin-2, pif-like, tyrosinase-like unigenes have the highest expression levels in the mantle tissue than the levels in the other tissues, indicating their primary functions for biomineralization process. This study can contribute to the understanding of the molecular mechanisms and the functional components of the proteins that involve the biomineralization process of the calcite foliated plates of *P. placenta*. The transcriptomic data generated in this study provide a basis for further studies of *P. placenta* genome. Moreover, the comparison of potential biomineralization genes also reveals the similarities and differences between shell formation matrix of different molluscan animals.

## Supplementary Information

Supplementary Information 1.

Supplementary Information 2.

Supplementary Information 3.

Supplementary Information 4.

Supplementary Information 5.

Supplementary Information 6.

Supplementary Information 7.

Supplementary Information 8.

Supplementary Information 9.

Supplementary Information 10.

Supplementary Information 11.

Supplementary Information 12.

Supplementary Information 13.

## Data Availability

All relevant data reported here are included in the main section of the manuscript or in the supplementary materials. All described materials are available upon request.

## Abbreviations

qRT-PCR: Quantitative real-time PCR
SSR: Simple sequence repeat
SNP: Single nucleotide polymorphisms
Indel: Insertion–deletion mutations
KOG: EuKaryotic Ortholog Groups
KEGG: Kyoto Encyclopedia of Genes and Genomes
GO: Gene Ontology
CDSs: Coding regions
nr: NCBI non-redundant protein sequences
Pfam: Protein family
SMPs: Shell matrix proteins
VWA: Von willebrand factor type A
FPKM: Fragments per kilobase of feature per million mapped reads
