# Supplementary material for: Transcriptome analysis of the bivalve Placuna placenta mantle reveals potential biomineralization-related genes
Source: Sci Rep. 2022 Mar 18;12:4743. doi: 10.1038/s41598-022-08610-5 (PMC8933548; doi:10.1038/s41598-022-08610-5)
Supplement: Supplementary file 1 — Supplementary Information 1. [file 41598_2022_8610_MOESM1_ESM.docx]

**Supporting Information listing**

Supplementary Excel S1: Extracellular region

Supplementary Excel S2: P and T category

Supplementary Excel S3: Wnt signaling and tyrosine metabolism pathways

Supplementary Excel S4: Hemokine signaling and leukocyte transendothelial migration pathways

Supplementary Excel S5: SNPs

Supplementary Excel S6: Indels

Supplementary Excel S7: SSR_primer

Supplementary Excel S8: Alignment results with genes potentially related to biomineralization in the mantle transcriptome of *P. placenta*

Supplementary Excel S9: Comparison with proteomic data of *C. gigas* shells

Supplementary Excel S10: Comparison of mantle transcriptomes of three scallops

Supplementary Excel S11: Pfam domain analysis from the *P. placenta*

Supplementary Figure S1: The distribution of Indel types

Supplementary Figure S2: Frequency distribution of SSRs based on motif sequence types

Supplementary Table S1: Primer sequences for qRT-PCR analysis
