# Supplementary material for: Transcriptome analysis of the bivalve Placuna placenta mantle reveals potential biomineralization-related genes
Source: Sci Rep. 2022 Mar 18;12:4743. doi: 10.1038/s41598-022-08610-5 (PMC8933548; doi:10.1038/s41598-022-08610-5)
Supplement: Supplementary file 2 — Supplementary Information 2. [file 41598_2022_8610_MOESM2_ESM.docx]

Table S1. Primer sequences for qRT-PCR analysis.

| **Unigene ID** | **Forward primer (5'to3')** | **Reverse primer (5'to3')** |
| --- | --- | --- |
| c76266 | GCGCTGAATATGTTGGGCTT | GGTAGCACGTACACCGATTG |
| c67461 | GTGGGCTGAAGCAATGAGAG | GCCATGTTCCTTCCTTGACC |
| c73086 | TGCATAAGCGAGCTGGAAAC | CTTTGCCGAGCGAAGTTGTA |
| c69385 | TGTGTGGACAGGACGGTAAA | CTCCGTTGGAAACAATGCCA |
| c81494 | GAAATGAACTGCGGACTGCT | AACTGCTCACACTCCTCCTC |
| c70548 | TAAGAAAGGCGGCAAAAAGA | ATCGTCCACTGGATTCTTCG |
| c84621 | TGAATGGGAACCACCCATGA | ACCTGCCACAAATCGAAACC |
| c66761 | ACAGCAGTAACCTCCGGAAA | ATCAAAGAGGCTGCACAAGG |
| c84941 | TGGAGATGTTCCGATCCGTT | GTCACTTGTCCCGCTTTGTT |
| c59513 | CGTGAGCAGACAACTGGATG | GAGAAGTGGTTTGGCACTGG |
| c73225 | AAGGTGTGTCACCCATCGAA | GACCTGGGCCATCATTTGTC |


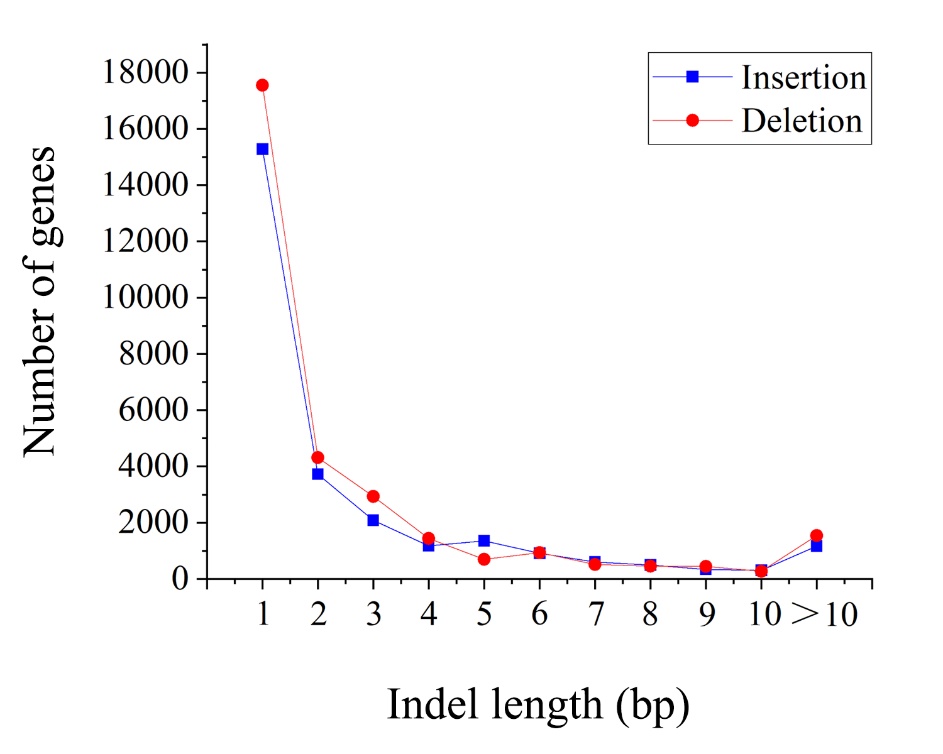


Fig. S1. The distribution of Indel types


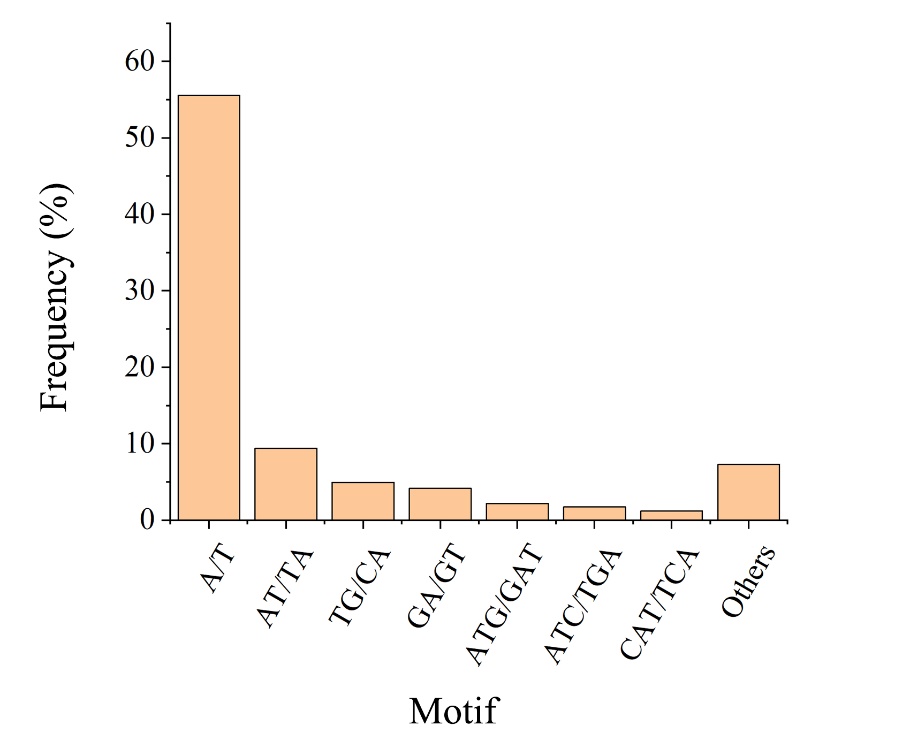


Fig. S2. Frequency distribution of SSRs based on motif sequence typ
